# The Development and Evaluation of Novel Patient Educational Material for a Variant of Uncertain Significance (VUS) Result in Hereditary Cancer Genes

**DOI:** 10.3390/curroncol31060256

**Published:** 2024-06-16

**Authors:** Deborah Cragun, Marleah Dean, David Baker, Meghan Kelley, Gillian Hooker, Anne Weidner, Paige Hunt, Tuya Pal

**Affiliations:** 1 College of Public Health, University of South Florida, Tampa, FL 33620, USA; 2 Department of Communication, University of South Florida, Tampa, FL 33620, USA; 3 Outcomes & Behavior Program, Moffitt Cancer Center, Tampa, FL 33612, USA; 4 Department of Medicine, Vanderbilt University Medical Center, Nashville, TN 37212, USA; 5 Morsani College of Medicine, University of South Florida, Tampa, FL 33620, USA

**Keywords:** ambiguity, cancer gene, family sharing, genetics, variant of uncertain significance, VUS

## Abstract

A Variant of Uncertain Significance (VUS) is a difference in the DNA sequence with uncertain consequences for gene function. A VUS in a hereditary cancer gene should not change medical care, yet some patients undergo medical procedures based on their VUS result, highlighting the unmet educational needs among patients and healthcare providers. To address this need, we developed, evaluated, and refined novel educational materials to explain that while VUS results do not change medical care, it remains important to share any personal or family history of cancer with family members given that their personal and family medical history can guide their cancer risk management. We began by reviewing the prior literature and transcripts from interviews with six individuals with a VUS result to identify content and design considerations to incorporate into educational materials. We then gathered feedback to improve materials via a focus group of multidisciplinary experts and multiple rounds of semi-structured interviews with individuals with a VUS result. Themes for how to improve content, visuals, and usefulness were used to refine the materials. In the final round of interviews with an additional 10 individuals with a VUS result, materials were described as relatable, useful, factual, and easy to navigate, and also increased their understanding of cancer gene VUS results.

## 1. Introduction

Approximately 5% of all colorectal cancers and 5 to 10% of breast cancers are caused by hereditary cancer predisposition syndromes that result from inheriting a germline pathogenic or likely pathogenic variant (GPV) in any one of several cancer risk genes [1,2]. Improvements in access to genetic testing and the ability to test for many genes simultaneously at a similar cost as testing for one or two genes have led to a higher number of patients identified as having a Variant of Uncertain Significance (VUS) result. Unlike GPVs, which are known to increase cancer risk, a VUS result has an uncertain impact on gene function and cancer risks. In a large study, 90% of VUS results were eventually downgraded to a negative result [3]. Based on several guidelines, including the American College of Medical Genetics (ACMG) and the National Comprehensive Cancer Network (NCCN), a VUS in a hereditary cancer gene should not change a patient’s medical management, and it is vital for patients to understand these guidelines to prevent unnecessary medical interventions [4,5].

While a VUS should not influence medical management, it remains important to discuss any personal and family history of cancer to guide cancer risk management among those with a VUS, similar to what is advised for those with negative genetic test results. For example, individuals who have a first-degree relative with certain types of cancer, such as breast or colorectal cancer, are at a higher risk for cancer themselves, and changes in cancer risk management, such as heightened cancer surveillance, may be warranted based on this familial risk [6,7,8,9,10].

While family history may justify increased cancer surveillance, misinterpretation of VUS results may result in risk-reducing surgeries in some patients [11]. This illustrates the need to educate both patients and healthcare providers about VUS results, while simultaneously using the opportunity to highlight the ongoing importance of considering familial cancer risks. Through this study, we sought to develop, evaluate, and refine novel educational materials to explain VUS results and educate individuals on how sharing information about a personal or family history of cancer remains useful as it can guide cancer risk management among family members.

## 2. Methods

We utilized an iterative process that began with an educational needs assessment to inform the development of VUS educational materials. This was followed by a three-step evaluation of the materials interspersed with several rounds of revisions. Our methods were determined to constitute an evaluation of educational materials by the University of South Florida’s Institutional Review Board (IRB) rather than human subjects research. Nevertheless, all individuals who provided feedback as part of our process received an informational sheet (similar to an informed consent document) and consented to reviewing the materials and answering questions while being recorded.

### 2.1. Educational Needs Assessment and Materials Development

The primary goal of our needs assessment was to identify common misconceptions, key information, or messages that might be useful in designing VUS educational materials. To achieve this goal, we reviewed the relevant VUS literature and analyzed de-identified interview transcripts from six women with a VUS in a hereditary breast cancer risk gene that had been conducted during a prior research effort to understand cancer risk management and genetic test results disclosure [12].

Themes and supporting examples from both the literature and interview transcripts were independently extracted by two team members (DC and MK) with expertise in health education and behavior. Findings were then combined, categorized, and discussed with the assistance of additional evaluation from team members, including experts in health communication, genetics, and anthropology. The team revised and recategorized themes until an agreement was reached. Team members subsequently used findings from the needs assessment to modify an existing audio/visual tool and to create additional educational materials.

### 2.2. Materials Evaluation

The three-step evaluation process included an assessment of materials based on criteria from an established evaluation rubric, followed by two rounds of feedback elicited from individuals with a VUS, as well as behavioral scientists and genetics experts who were not involved in development. First, team members used the Centers for Disease Control and Prevention (CDC) Clear Communication Index (CCI) Score Sheet [13] to assess the materials’ main messages, language level, information design, and behavioral recommendations [13]. A score of 1 was given when the material met criteria for each of the various aspects in the checklist. A score of 0 was assigned when the material did not meet the criteria. Scores of 90 and above were considered passing.

In addition to evaluating the materials using the CCI, team members solicited two rounds of feedback. The first round of feedback on the materials was elicited during semi-structured interviews and a focus group. Semi-structured interviews were conducted with two individuals from the Inherited Cancer Registry (ICARE) [14] who were confirmed to have a VUS and with one genetic counselor recruited from the National Society of Genetic Counselors (NSGC) website. A subsequent focus group to collect additional feedback was conducted among four individuals, including a genetic counselor, clinical geneticist, and two PhD-level scientists with expertise in public health, behavioral science, and hereditary cancer. The first round of feedback was used to improve the acceptability, clarity, and appropriateness of the educational materials, and revisions were made (as outlined in Section 3. Results and Refinement of Educational Materials). Subsequently, to evaluate the revisions to the materials, a second round of feedback was elicited during interviews with 10 different individuals from ICARE who were confirmed to have a VUS result in a cancer risk gene. This additional feedback supported earlier changes made to the materials and was used to finalize and provide preliminary support for the potential efficacy of the materials, as described in Section 3. Results and Refinement of Educational Materials.

Interviews for both rounds of feedback, as well as the focus group, were conducted via virtual video conferencing to allow participants to review the materials and describe their impressions in real-time. After participants provided initial feedback throughout the materials review, questions were asked to elicit more feedback on the material’s understandability, appropriateness, and acceptability. Specific to the second round of feedback, additional questions were asked to assess the usefulness of materials in sharing information with family members and to determine if participants’ perceptions changed regarding a VUS or cancer risks. Individual interviews and the focus group were recorded, and responses were coded and summarized into Microsoft Excel documents that included a list of recommendations for what to change or fix, as well as other emergent themes. Specific to the second round of feedback, themes were further organized into categories and subcategories with several exemplary quotes being extracted from interview recordings to illustrate key findings.

## 3. Results and Refinement of Educational Materials

This section summarizes findings from each step in the development and subsequent evaluation process and provides a few examples of changes and refinements made to the materials.

### 3.1. Findings from the Educational Needs Assessment and Materials Development

First, no published research studies were identified that detailed the development or evaluation of educational materials designed specifically for patients with a VUS in a cancer risk gene. However, an online tool was identified to help family members of a patient with a VUS understand VUS results, with the goal of recruiting family members into variant interpretation research studies [15]. This online tool did not describe the development of their information, nor did it include materials designed to increase family sharing of cancer risk information and cancer screening.

In general, the literature review findings highlighted difficulties patients have in understanding their VUS result and their struggle to accept the lack of healthcare recommendations [6,7,8,9,10]. Studies report how some non-genetics providers have misinterpreted VUS results and, in some cases, even recommended inappropriate medical interventions, such as a prophylactic mastectomy, for VUS results that were eventually reclassified as benign [16,17]. Patients also tend to misinterpret their own VUS results and may communicate inappropriate or confusing messages to family members [18,19]. When patients’ explanations are less reassuring and clear, it is more likely that their family members will perceive cancer in the family as hereditary, and the VUS as being associated with higher cancer risks [20].

Second, findings from our analysis of de-identified interview transcripts were also used to develop VUS educational materials (see Supplemental Table S1 for characteristics of the six participants with a VUS who had consented previously). Some participants expressed initial confusion over their VUS results, which then turned into frustration due to the lack of impact these results have on medical management. In contrast, other patients seemed to understand that although the results do not change anything now, it might be important for family members to know about a VUS in the event that more information is found about the VUS in the future. These findings informed messages included in our “VUS Video: Understanding your VUS Test Result” and “Key Messages to Share (VUS/Family History)” educational materials.

When prompted to offer advice to other individuals who might receive a VUS result, one participant recommended disseminating risk information throughout the family in steps, starting with the family members perceived to be the most supportive. Although one patient expressed that sharing VUS results should be considered a necessity, another described how they will only share their VUS with family members if it is reclassified as a GPV. Finally, interview transcripts revealed that hearing about others’ experiences may be helpful. As such, we created educational materials entitled “Others’ Experiences with VUS Test Results” and “Experiences with Sharing Cancer Family History.”

Together the literature review findings and the analysis of interview transcripts with patients who have a VUS provided a rationale for developing VUS educational materials (see column entitled “Rationale to Inform Intervention Components” in Table 1) and informed the content of materials (see Table 1 for descriptions of the materials and screenshots).

### 3.2. Evaluation: Clear Communication Index (CCI) Results and Revision

When evaluated, the educational materials initially scored 60% according to the CCI. Reasons for point deductions include the materials having three main messages as opposed to a single main message, and the call-to-action was listed in the passive voice. Points were also lost because some unfamiliar terms were only defined but not further explained and because some numbers were presented in only one way; for example, lifetime risks for cancer in various scenarios were listed only as percentages.

The materials scored high in clarity and communication because they included a call-to-action promoting family sharing of cancer risk information, used whole numbers when describing the efficacy of mammograms and colonoscopies, and explained how a family history of cancer can increase cancer risks even when no genetic cause is found. Points were also assigned because materials acknowledged the benefits associated with the recommended behavior.

Several changes were incorporated into the materials to improve the CCI, resulting in a score of 90%. This score was considered to be acceptable since some of the points were lost because risks of mammograms and colonoscopies were not explained to keep these respective handouts to one page. Points were also lost because the intervention included materials about VUS results as well as cancer screening based on family history, and the landing page helped guide individuals to the different resources rather than containing the main messages for each of the key materials. Points were not deducted for following the requests of participants to add a number to one of the handouts. This number was stated in the video and was included in a visual there, but the audience believed it was straightforward and enabled the handout to remain within one page.

### 3.3. Evaluation: Feedback and Materials Refinement (Round One)

Both of the patients and the genetic counselor who provided initial feedback reported overall satisfaction with the materials, but they also offered a few minor critiques concerning the language used and the design of the materials. For example, one patient shared the importance of having a primary care physician organize, interpret, and manage screenings, given that it is difficult for patients to be solely responsible. Another patient expressed some initial confusion about the VUS result, but after viewing our VUS educational video, she accurately described why her VUS did not explain her strong family history of cancer. The genetic counselor’s feedback focused on the benefit of educational materials regarding modifiable risk factors for cancer (e.g., tobacco use, excessive sun exposure), which they suggested could be incorporated into the video’s “Learn More” sections.

Focus group members appreciated the simple language used and overall material intent. A large portion of the focus group discussion centered on the decisional balance handout that listed “pros” and “cons” of sharing information about a family history of cancer. The group liked the use of this same decisional balance approach for material on sharing VUS results, because VUS results are not clinically actionable; however, they were concerned that the “cons” of sharing information about a family history of cancer may create more hesitancy in communicating cancer risk information. Given that a family history of certain cancers may change medical care for close relatives, the group felt it was important to promote family sharing while still acknowledging the concerns people may have.

Multiple changes were made to address both minor and major constructive feedback from these interviews and the focus group (see Supplemental Table S2). As one example, the “Sharing family history information” handout was made into a checklist of reasons for sharing, and the considerations (or potential cons) were included as part of others’ experiences illustrating ways to respond to family member reactions.

### 3.4. Evaluation: Results and Materials Refinement (Round Two)

The second round of feedback primarily uncovered minor improvements or edits from the 10 interviewees in round two of the evaluation (see Supplemental Table S3 for participant demographics). The video format was preferred by five participants, though three preferred the handout format (Supplemental Figure S1). All 10 interviewees commented on how the material was “concise”, “succinct”, “straight-to-the-point”, and “only tells you what you need to know”.

Overall, interview participants described the material as effective at improving their understanding about their VUS results. As one participant shared, “…These materials would have changed my perception and knowledge of a VUS when I first got my result”. Furthermore, some interviewees stated that the materials clarified their confusion when they received their initial VUS results. One participant said “It’s a clear description of a VUS that clears up the confusion and ambiguity associated with a VUS result”. Another interviewee shared “I have been confused as to what all that [referring to their VUS result] meant. I did not know that and just learned that from this [referring to VUS materials]”.

Interview participants also described the VUS materials as “empowering” and provided clear action steps, which reduced fear. For example, one participant said, “I now know what to do and that makes it not so scary”. Additionally, several interviewees noted the different reasons provided in the “Sharing your family’s history of cancer” handout and the quotes in the “Others’ experiences” section reflected how they felt or was similar to their own situation when talking with family members, with one participant saying “[The patient experience where] he mentioned a family history of cancer three times and his brother finally got his colonoscopy, it sounds exactly like my experiences”.

Participants reported that the materials would be helpful in sharing information about a VUS result, as well as sharing information about one’s family history of cancer. For instance, one participant shared, “I think people really struggle with what to say, and if you spoon-feed them, then that’s going to make it so much easier for them to share this information”. Indeed, several interviewees expressed that they wished the materials were more persuasive in sharing a VUS result; however, the main purpose of the intervention was to make it clear that sharing a VUS result is an individual preference, and it does not impact their family’s medical care (unlike a GPV). One participant noted that we achieved this goal by stating the material is “pretty unbiased” and “doesn’t seem like it’s trying to push you in any direction”. Last, all 10 interviewees indicated they would share these materials.

Although the feedback was predominantly positive (see Supplemental Table S4), there were some constructive comments (see Supplemental Table S5). For example, some interviewees expressed negative feelings evoked by a graphic of a scale (or balance) that was used to represent the weighing of the pros and cons on the “Deciding about Sharing Your Genetic Test Result” handout. That image was subsequently replaced by another image of a human silhouette shrugging. Furthermore, interview participants had mixed feelings on the intervention’s color scheme.

Several minor changes were implemented based on feedback from round two (noted in Supplemental Table S5). For example, we added guidance on which family members are the most important to talk to and offer additional “action items” to give patients directions/next steps they can take after learning about their VUS result. Due to the inability to garner consensus, the research team voted to keep the original color scheme.

## 4. Discussion

Through our study, we describe a systematic multi-step approach to the development, evaluation, and refinement of materials designed to educate patients and healthcare providers that a VUS result does not change medical care yet it remains important to share personal and family cancer information with family members. Comments from the final round of evaluation suggest these materials can fulfill the need to help patients with VUS results understand their results [21] and improve communication about cancer risks, as well as screening and prevention options between and among family members [6,7,8,9,10]. Additionally, our educational materials not only utilize but also extend prior research in multiple ways.

First, we addressed known misconceptions leading to inappropriate medical decisions. Patients struggle to understand their VUS results and thus often believe their cancer risks remain high because of the result [19,22]. The ambiguity inherent in a VUS result means that it should not be used to alter medical care. However, the lack of clear action steps also creates uncertainty for patients and healthcare providers [11,23,48], which can result in inappropriate medical decisions [21,25]. Our materials emphasized that a VUS result should not be used to change medical care, but rather medical management should be based on a family history of cancer. The potential efficacy of these materials to educate patients about VUS results for cancer genes is evidenced by the respondents’ ability to relay back messaging that the VUS result would not impact medical management screening, treatments, and other health risks.

Second, we included two decision-making support resources to help patients who have a VUS result—(1) to consider the pros and cons of sharing their VUS result and (2) to identify their own reasons for sharing their personal and family history of cancer with family members. These two separate resources should help distinguish between these decisions and reflect how the benefits of sharing a VUS result are less clear than the benefits of sharing family history information. Thus, sharing a VUS result should be based on the patients’ preferences when considering pros and cons together as part of a decisional balance exercise. In contrast, considering the benefits of sharing a family history of cancer first and then reviewing patients’ experiences (for anyone who has concerns) should help motivate participants to share this information, while also addressing ways to overcome the reservations they may have (illustrated by patients’ experiences). Participants throughout the evaluation believed that these resources would be useful and extant research also notes that sharing genetic test results with family members is an effective coping strategy [24].

Third, as alluded to above, we included patients’ experiences with sharing VUS results, as well as sharing their family history of cancer. Integrating patient narratives such as quotes provides psychosocial information that resonates with patients, helping them to process and manage information [44] and may motivate them to adopt certain health behaviors [45]. Specific quotes that illustrate different coping responses can demonstrate appropriate cancer risk management decisions and forecast possible reactions that family members may have if patients share information [46,49]. A novel aspect of this section in our intervention is the use of real patients’ quotes from extant research [44], as well as interviews from our prior study. Importantly, our participants who evaluated the educational materials reported that the patients’ quotes resonated with their personal experiences when sharing their VUS result and/or family history of cancer with family members.

### Strengths and Limitations

Our study has several strengths, including the purposeful utilization of the relevant literature and patients’ VUS experiences and a clear demonstration in Table 1 explicating how we developed our intervention’s educational materials. As noted earlier, no research studies were found explaining not only the development, but also the evaluation of educational materials for patients with a VUS in a cancer risk gene. Another strength is the iterative evaluation process. We sought out not only feedback from patients with a VUS result, but also other important stakeholders including experts in genetics and public health and behavioral science. Despite these strengths, there remain some limitations including the fact that the participants in this study had already shared information about their VUS result and/or family history of cancer; thus, we may be missing perspectives from patients who are hesitant or resistant to sharing. Second, the views may not be representative given that participants included only one male and three individuals who identified as racial/ethnic minorities.

## 5. Conclusions

This study reports on the development, evaluation, and refinement of novel educational materials that explain that VUS results should not change medical care and educates patients on how sharing information about a personal or family history of cancer may be useful to family members. By addressing known misconceptions about VUS results, providing decision making support for sharing information, and integrating patients’ experiences, the goal of our educational intervention is to help patients with VUS results manage their uncertainty, understand their cancer risks, and facilitate communication with family members. While recognizing that these educational materials were designed for patients, we believe that these materials may also assist healthcare providers in explaining VUS results and promote appropriate cancer risk management decisions. Future studies are now needed to test whether these materials are useful for ensuring accurate information is shared with family members.

## Figures and Tables

**Table 1:** Variant of Uncertain Significance (VUS) educational intervention components.

Educational Components Brief Description	Intervention Screenshots Visual Examples	Rationale to Inform Intervention Components Literature Review and Existing Interviews
**VUS/Family History Website: Overview** 5 min video—Understanding your VUS test result Frequently Asked Questions (FAQs) Deciding whether to share a VUS test result Empowering your family with cancer risk information Support resources and others’ experiences **VUS Video: Understanding your VUS Test Result** Discuss different types of test results Covers why family members do not usually need clinical testing for a VUS Explains how a family history of cancer may change medical care for individuals and their family members	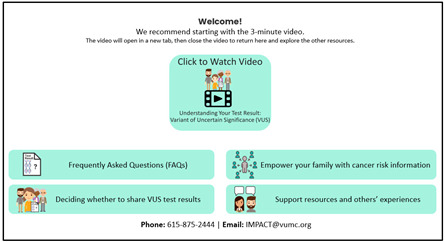 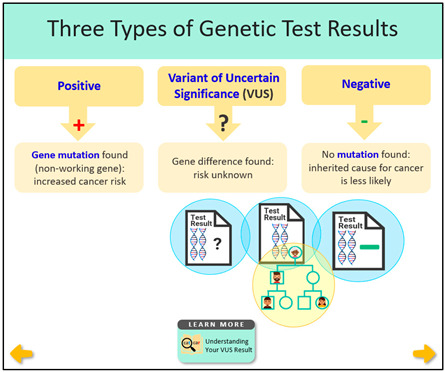	**Literature Review Findings** Individuals with VUS test results need educational materials to understand their results [21]. [*Introductory video and FAQs provide basic information and key messages.*] People with VUS results believe their cancer risks remain high [19,22]. [*Key message: Cancer risks depend on personal and family history of cancer.*] Those with VUS results experience uncertainty and confusion [18,23,24], which can result in inappropriate medical decisions [21,25]. [*Key messages: VUS result should NOT be used to change medical care; medical management should be based on cancer family history.*] **Existing Patient Interviews** “Maybe like a video or something would have helped.” (Female 1, *BRCA2* VUS) [*Reason video was developed.*] “Finding out that it wasn’t positive made it easier for me to decide, because if it was positive, I was just going to do the mastectomy” (Female 1, *BRCA2* VUS). [*Key message: a VUS is different than a positive test result.*] “The way she [genetic counselor] explained it and broke everything down for my husband and myself just made me feel better, that basically it could end up meaning nothing…they can let me know if they do find something with it” (Female 8, *BRCA1* VUS). [*Key message: When VUS results are reclassified, most are negative, meaning they do not increase cancer risk.*] “I guess feeling confident and knowing what it means yourself helps you to explain it to other people” (Female 1, *BRCA2* VUS). [*Improved understanding may increase confidence in sharing correct information with family or other healthcare providers*.]
**Decision Making:** **Sharing your Genetic Test Result** Downloadable PDF Handout Acknowledges that sharing one’s VUS test result is not always easy Provides reasons for why people may decide to share their VUS test result Can be printed or completed electronically Shares a link to resources for sharing VUS test results	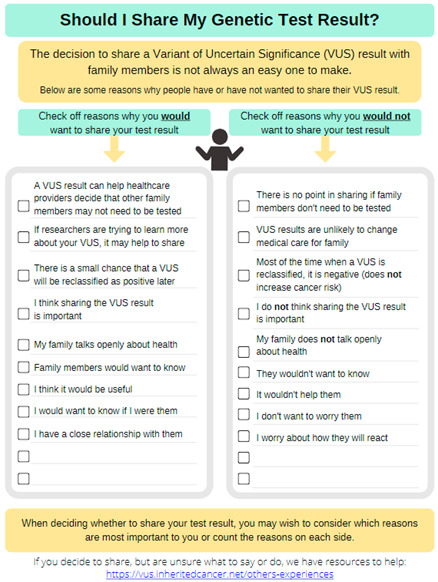	**Literature Review Findings** Effective coping strategies include seeking information and sharing genetic test results with family members [24]. [*Justifies the inclusion of a section on sharing information with family*.] Some patients do disclose their VUS results to family members [26,27,28]. [*Materials that are easy to share may help ensure that family members receive appropriate messages.*] Some people prefer not to share VUS results to avoid their family members misunderstanding the results and creating a ‘false alarm’ [26]. [*Reasons for not sharing VUS results are useful to consider*]. Decisions in which the benefits are uncertain or influenced by individuals’ preferences should be made by each person after considering the pros and cons and clarifying which of these are most important or relevant for them [29]. [*A decisional balance worksheet on sharing VUS test results was created because a VUS is not actionable for family members and benefits of sharing do not necessarily outweigh risks.*] **Existing Patient Interviews** “She [genetic counselor] didn’t recommend that [i.e., sharing test result with family] She said it was our decision whether we should share with the kids or not” (Female 2, *BRCA2* VUS). “There’s nothing you can do, nothing they [family members] can do, it doesn’t mean anything right now” (Female 8, *BRCA1* VUS). “We actually have a copy of the genetic testing in with our will and trust. And if we both pass at the same time then that information will become available…But we chose to not share it with them unless we get an update in the future that says, yeah this is an important thing to share” (Female 2, *BRCA2* VUS).
**VUS Single Page Handout** What is a Variant of Uncertain Significance? Should I be tested for a VUS found in my family? What does this mean for my cancer risk? What can I do to lower my cancer risk?	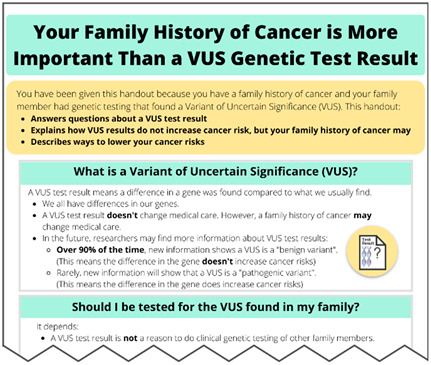	**Literature Review Findings** Family members report print materials are helpful in the context of positive results [30]. [*Printable handouts may also be helpful in the context of a VUS result.*] **Existing Patient Interviews** “My husband and I looked at it [take-home educational info about the test result] on numerous occasions too, so I do feel like having something more than just the results to go home with, something else that talks about genetics [would be] helpful” (Female 3, *BRCA2* VUS). [*Emphasizes the value of handouts that can be printed or downloaded and easily shared*.]
**Decision Making:** **Sharing your Family’s History of Cancer** Downloadable PDF Handout Provides common reasons for why people share Includes space to add other reasons	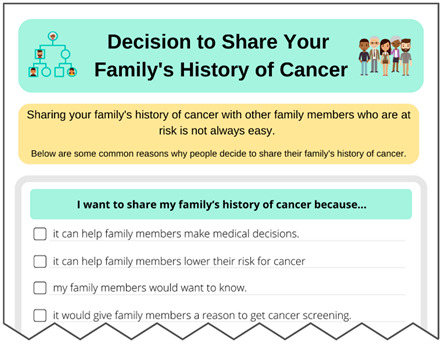	**Literature Review Findings** Even in the absence of a positive genetic test result, close relatives of someone with certain cancers have a higher risk for cancer, and they may need and benefit from additional or earlier cancer screening [31,32,33,34]. When the benefits of a particular action outweigh the risks (which is often the case for cancer screening for those with an increased risk), people are more likely to take action if they identify their own motivations rather than being told what to do [35]. [*This worksheet was not framed as decisional balance because family history may change medical care and can help relatives. This may also help differentiate the decision to share a family’s history of cancer from the decision to share a VUS result.*] **Existing Patient Interviews** NA
**Key Messages to Share (VUS/Family History)** Downloadable PDF Handout Starting the conversation Family members’ possible reactions Key messages to share about the family history of cancer	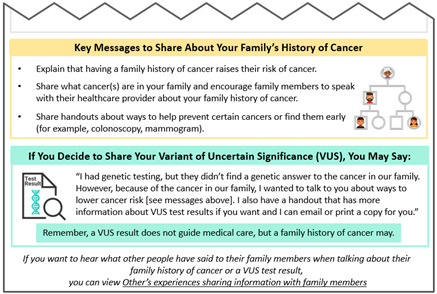	**Literature Review Findings** In order to disclose genetic test results, patients need to feel prepared [36]. [*Modeling key messages may help individuals feel more prepared*.] Key messages that are simplified increase recall of information, regardless of health literacy level [37]. Visuals can reinforce the key messages and aid understanding for people with lower literacy [37,38,39]. **Existing Patient Interviews** NA
**Planning Guide for Sharing Family History** Downloadable PDF Handout Initial sharing—Who? When? How? Follow-up with family members—Who and when?	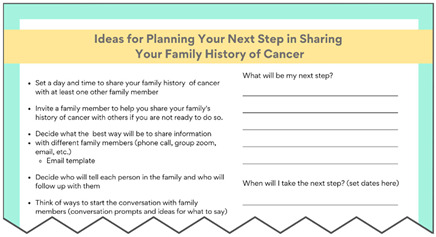	**Literature Review Findings** Detailed action planning about when and how to perform a behavior is more likely to lead to completion of the behavior [40]. [*Planning may help individuals share information and follow-up with family members*.] Research on follow-up with family members is limited. However, some family members forget or misremember the genetic testing information that was shared with them [41]. [*Materials explain the value of making sure family members remember and understand the information. This guide includes space to create a plan for both initial sharing and follow-up with family*.] **Existing Patient Interviews** NA
**Cancer Family Health History Questionnaire** Questionnaire to fill out regarding: Personal health history of cancer Biological family history—helps prompt them to consider family members on both the father’s side and mother’s side and ask about the cancer type and age of diagnosis	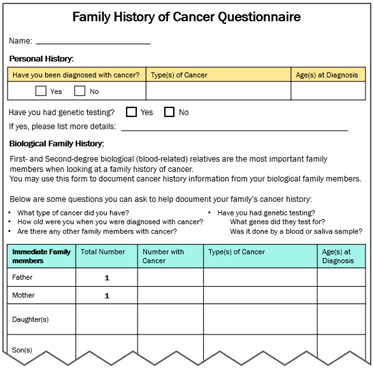	**Literature Review Findings** Women with a family history of breast cancer underwent more mammograms than those with no family history [42]. First-degree relatives were nearly twice as likely to have had a colonoscopy as other relatives [34]. [*This suggests raising awareness of family history (especially among first-degree relatives) might prompt cancer screening*.] **Existing Patient Interviews** NA
**Breast/Colon Cancer Handouts and Videos** Breast/colon cancer risks depend on family history Finding cancer early improves outcomes Breast/colon cancer screening saves lives	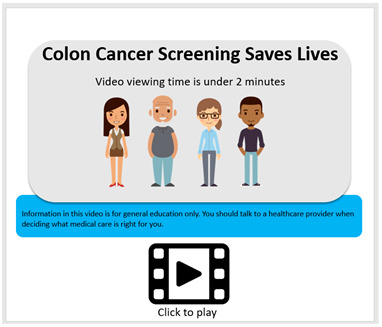	**Literature Review Findings** Materials were informed by Family CARE visuals [43] that used a fear appeal and efficacy messaging that, when combined with motivational interviewing, proved effective at increasing colonoscopy among those with an affected first-degree relative [7]. [*Illustrates higher cancer risks for those with a mother, father, brother, or sister with breast or colon cancer (threat message) and informs about the effectiveness of cancer screening (efficacy message).*] **Existing Patient Interviews** “You know we’ve had some concerns, she [first-degree relative] had severe colon cancer, and we heard that can run in families… our kids have all had early colonoscopies just to be safe.” (Female 2, *BRCA2* VUS) “I kind of tell them about do a lot of prevention screening, that maybe not genetic test, but just do a lot of prevention screening because my diagnosis was in the early stage” (Female 4, *BRCA1* VUS).
**Others’ Experiences** **… with VUS Test Results** Initial reactions to a VUS result What people have done if they are still worried about a VUS test result Planning cancer screening based on family history Managing cancer screening **… with Sharing Cancer Family History** Sharing family history Deciding not to share VUS test result Plan to share a VUS test result in the future Decided to share a VUS test result	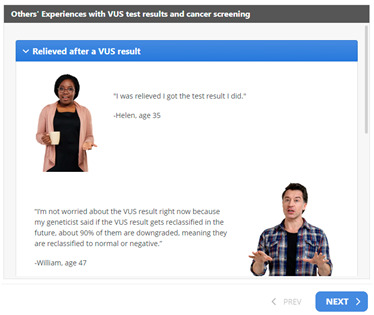 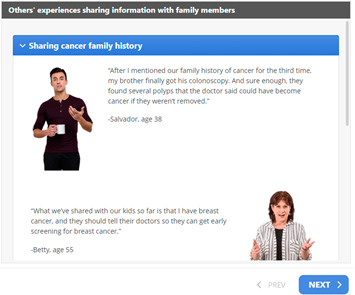	**Literature Review Findings** Integrating patient narratives provides psychosocial information that resonates with patients, helping them process and manage information [44] and may motivate them to adopt certain health behaviors [45]. [*Quotes that illustrated coping responses have the potential to positively influence individuals and can demonstrate appropriate actions to take*.] Anticipated reactions of family members influence if individuals share their genetic test results in general [46,47]. [*Some of the experiences are about sharing information with family and how their family members reacted. Many are based on patient quotes from existing or follow-up interviews*.] **Existing Patient Interviews** “It’s good. At least it’s not positive” (Female 4, *BRCA1* VUS) [*Although relief is just one reaction, we included an array of emotions/reactions (e.g., disappointment, ongoing worry, confusion, etc.)*]. “It doesn’t mean I have hereditary cancer, so I feel pretty much the same. I mean, I know it still could be something. But right now, I haven’t heard anything more so I’m not too worried.” (Female 1, *BRCA2* VUS) “I told my family the result was OK, so they don’t need testing because a VUS won’t change their care.” (Female 4, *BRCA1* VUS). “What we’ve shared with our kids so far is I have breast cancer and they should tell their doctors so they can get early screening (Female 2, *BRCA2* VUS). “After I mentioned our family history of cancer for the third time, my brother finally got his colonoscopy, and sure enough, they found polyps that the doctor said could have become cancer if they weren’t taken out”. [*Quote was created, but two participants who reviewed the materials said this resonated with their own experience.*]

## Data Availability

The datasets presented in this article are not readily available because the data are part of an ongoing study. Requests to access the datasets should be directed to the corresponding author.

## Supplementary Materials

The following supporting information can be downloaded at: https://www.mdpi.com/article/10.3390/curroncol31060256/s1, Figure S1: Material format preference from second round of interviews; Table S1: Demographics of the six interview participants from the needs assessment; Table S2: Feedback from the needs assessment interviews and focus group; Table S3: Demographics of interview participants from materials’ validation; Table S4: Positive feedback organized by category and subcategory with supportive illustrative quotes; Table S5: Constructive feedback organized by category and subcategory with supportive illustrative quotes and description of changes made.

## Abbreviations

VUS	Variant of Uncertain Significance
IRB	Institutional Review Board
CDC	Centers for Disease Control and Prevention
CCI	Clear Communication Index
ICARE	Inherited Cancer Registry
NSGC	National Society of Genetic Counselors
COM-B	Capability, Opportunity, Motivation-Behavior
GPV	Germline Pathogenic or Likely Pathogenic Variant
