# Supplementary material for: The Development and Evaluation of Novel Patient Educational Material for a Variant of Uncertain Significance (VUS) Result in Hereditary Cancer Genes
Source: Curr Oncol. 2024 Jun 16;31(6):3361–78. doi: 10.3390/curroncol31060256 (PMC11202617; doi:10.3390/curroncol31060256)
Supplement: Supplementary file 1 [file curroncol-31-00256-s001.zip › Supplemental Table S1.pdf]

Supplemental Table S1. Demographics of the six interview participants from the needs assessment.

ID	Age	Sex	Gene	Result [1]	Race & Ethnicity [2]	Personal History of Cancer
ICARE 1	36	Female	*BRCA2*	VUS	NHW	None
ICARE 2	67	Female	*BRCA2*	VUS	NHW	None
ICARE 3	47	Female	*BRCA1*	VUS	Asian	Breast
ICARE 4	50	Female	*BRCA2*	VUS	NHW	Breast. Skin
ICARE 5	67	Female	*BRCA2*	VUS	NHW	Thyroid, Colon, Skin
ICARE 6	42	Female	*BRCA1*	VUS	NHW	Colon

[1] Variant of uncertain significance (VUS)
[2] Non-Hispanic White (NHW)
