# Supplementary material for: The Development and Evaluation of Novel Patient Educational Material for a Variant of Uncertain Significance (VUS) Result in Hereditary Cancer Genes
Source: Curr Oncol. 2024 Jun 16;31(6):3361–78. doi: 10.3390/curroncol31060256 (PMC11202617; doi:10.3390/curroncol31060256)
Supplement: Supplementary file 1 [file curroncol-31-00256-s001.zip › Supplemental Table S2.pdf]

Supplemental Table S2. Feedback from the needs assessment interviews and focus group.

	VUS Video	VUS Single Page Handout	Breast/Colon Cancer Video and Handout	Family Sharing Aids	Website FAQ
Healthcare Provider 1	Gene difference found: unknown risk" make it clear that we don't know if there's a risk or not, not that we don't know what the risk is<br><br>Suggested including a slide about modifiable risk factors with cancer – smoking, diet – as lots of patients ask about these types of factors	Important to mention family members might need their own testing, especially if best testable in the family wasn't	Consider putting references on video<br><br>[In regard to the title of the breast cancer risk slide] "Breast cancer risk depends on a number of factors including family history"<br><br>Again, want to know modifiable factors.<br><br>[In regard to slide about survival if cancer is caught early versus later] What does survive mean? Instead say what number of breast cancers are curable when found early vs not	Consider saying: "Even though my genetic testing did not find any mutations, we still have a higher risk to develop cancer due to our family history."	"What Information Should I Share?" should come before, "Why Should I Share?"<br><br>More family members are being tested for VUSs now, so language might be too strong if saying this happens infrequently
Patient 1		The font could be bigger	Felt everything was clear/ not confusing	Did cover things that were said in participant's personal experience	
Patient 2	Suggested a slide about follow-up on genetic testing result, whether it is to contact their testing provider or the lab that ran their test<br><br>Important to include information to check back on test results every couple of years<br><br>Was provided a patient letter about VUS results. Felt the letter was very jargon-heavy and unhelpful, or confusing for those who aren't used to		Felt the slides about death rates if cancer found early/ late were extremely impactful. Felt they were understandable<br><br>Suggested having a slide dedicated to the importance of having a PCP or another healthcare provider to manage your care for you if you do need more screening.		"Make sure to define VUS at the top of the page."<br><br>"Does baby step into the bigger questions, easy to follow"

	medical terms. Said the materials were much easier to understand in comparison				
Focus Group	PhD Health Ed.: Is there any slide that explains what it means for a gene to be or not be working properly?<br>"Don't know if this change is important for your risk." Be a little more explicit with the sentence analogy and how it ties back to VUS<br>"How does genetic testing work" as a heading for one of the slides doesn't match with the content of the slide<br>Genetic Counselor 1: Make sure to define VUS in the narration of the first slide<br>Genetic Counselor 2: Mimicking the language that patients will see on test results is important as patients will often receive results before their doctors.<br><br>Make a slide or mention of how humans are all different and that most genetic changes are normal variation – everybody's DNA is different from everybody's DNA and we can find those changes with genetic testing<br><br>Normalize sharing genetic test results – perhaps explain the concept of the best testable		MD Geneticist: These sheets could be generalized to all cancers	There shouldn't be a pros/cons or decisional balance for sharing genetic test results or a strong family history of cancer<br><br>Work on supportive material on how to share results rather than giving a decisional balance to sharing or not.<br>Don't talk about pros and cons just go straight into considerations for sharing.<br>Also must be very distinct with language: use may not will in regard to risks, sharing<br>Explain why negative or VUS results in a family with a strong history of cancer aren't true negatives.<br>Try and frame the positives of sharing, especially as a, "did you know."	
