# Supplementary material for: The Development and Evaluation of Novel Patient Educational Material for a Variant of Uncertain Significance (VUS) Result in Hereditary Cancer Genes
Source: Curr Oncol. 2024 Jun 16;31(6):3361–78. doi: 10.3390/curroncol31060256 (PMC11202617; doi:10.3390/curroncol31060256)
Supplement: Supplementary file 1 [file curroncol-31-00256-s001.zip › Supplemental Table S3.pdf]

Supplemental Table S3. Demographics of interview participants from materials' validation.

ID	Age	Sex	Gene	Result [1]	Race & Ethnicity [2]	Personal History of Cancer
ICARE 7	37	Female	*BRCA2*	VUS	NHW	Breast
ICARE 8	35	Male	*MSH3, RAD51C*	VUS	NHW	None
ICARE 9	41	Female	*ATM, CHEK2*	VUS	NHW	None
ICARE 10	61	Female	*CDKN2A, RAD51D*	VUS	Black	Breast
ICARE 11	55	Female	*MSH6*	VUS	NHW	Breast
ICARE 12	35	Female	*BARD1*	VUS	NHW	None
ICARE 13	43	Female	*MUTYH, MUTYH*	VUS	Black	None
ICARE 14	69	Female	*CHEK2*	VUS	NHW	Breast
ICARE 15	55	Female	*APC*	VUS	Black	None
ICARE 16	48	Female	*PALB2*	VUS	NHW	None

[1] Variant of uncertain significance (VUS)
[2] Non-Hispanic White (NHW)
