# Supplementary material for: The Development and Evaluation of Novel Patient Educational Material for a Variant of Uncertain Significance (VUS) Result in Hereditary Cancer Genes
Source: Curr Oncol. 2024 Jun 16;31(6):3361–78. doi: 10.3390/curroncol31060256 (PMC11202617; doi:10.3390/curroncol31060256)
Supplement: Supplementary file 1 [file curroncol-31-00256-s001.zip › Supplemental Table S4.pdf]

Table S4. Positive feedback organized by category and subcategory with supportive illustrative quotes.

Category	Subcategory	Illustrative Quotes
**Content**	Concise, clear, and plain language	"For somebody who may be hearing about a VUS for the first time, I think this breaks it down in a really simplistic way." <br>"A summary sheet like [the VUS handout] would definitely help because you can't remember everything that genetic counselor is telling you. This boils it down, and it's helping me right now."
	Comprehensive	"[The handout on cancer risks] was precise and to the point, and I had everything I needed in one place."
**Education**	Good explanation of a VUS result	"[The introductory VUS video] was helpful honestly. The unspecified test results, I have that, so I've been confused about what all that really meant. So, this was good. And to see that most of those come back negative when they're retested was great information." <br>"I think that [the suggestion for how to explain a VUS result in the handout for conversations] is good… because it kinda starts off saying I had the testing, but it did not answer the question of why I have cancer. So, it reiterates the first couple of handouts."
	Conveyed importance of a family history of cancer	"[The VUS handout] highlights right up front that your family history is more important, so that's a good thing." <br>"This one statement [in the checklist of reasons to share a family history], 'cancer is serious and can be prevented', I think a lot of people forget that."
**Visuals**	Formatting	"Color scheme is good, everything reads well, it's not too dense, it's on a single page." <br>"I like [how the bolding of words and phrases] highlights and helps find what I care about… It directs attention."
	Graphics	"If you have colon cancer, and you have a family member who is not wanting to get screened for colon cancer for whatever reason, this [graphic on the colon cancer handout] would be an image I would show them."
	Accessibility	"I would actually post this [video on cancer risks] on social media or something… I think this is a sharable video."
**Usefulness**	Would make it easier to share information about cancer	"If I didn't have a family member I talk openly about [cancer with], and I wanted to give them more information, I would send them this video." <br>"I would have used [the planning guide for sharing with family members] had I had it [when first learned of VUS result]… I probably would have brought it with me to my doctor as well, just because it would help me keep track."
	Encourages sharing a family history of cancer	"[The checklist] does it's job if someone's struggling to share [their family history]." <br>"One of these reasons [on the checklist] I particularly like, it says 'Get support or advice from family'… Once [my family members] all started talking about our cancer and our screenings, [we] got lots of support from all sides of the family. I think that would be beneficial in helping anyone who's trying to decide whether to share or not to realize that they'd likely get support."
	Would have been helpful when	"I remember coming out of it kinda a relief that I got the [VUS] result I did. But also again, what do I do now type tying, like what are the next steps? So I think this is a good way to help somebody make that decision of 'hey, what are the next steps with this kind of result.'"

	receiving a VUS result	"This is great! If I had got this [VUS handout] with my test results that would have been awesome!... I wish I could get a handout like this for every test I take."
	Provided guidance on deciding to share a VUS result	"I'm pretty decisive with my family and decisive that they would want to know everything, so I wouldn't have much need for [the VUS sharing decision aid] myself. But I guess if people are worried, it seems to me [the decision aid] is pretty unbiased, doesn't seem like it's trying to push you in any direction."
		"It actually helped me looking at [the VUS decision aid], made me want to share the reasons like I did initially with my mother and father... I do like at the bottom that it says if you're undecided that there would be resources there, something that I could click on and get more information."
**Empowerment**	Increase efficacy	"Everybody takes bad news different ways, if you've got family members that are extremely private or stick their head in the sand when something bad happens, this [the sharing information with family members handout] has concrete steps to deal with them. So this could make the patients feel empowered, and that's what's really most important."
		"[The 'Share Information, Save Lives' handouts] kinda summarized everything… the risk, how to improve your outcomes, how to take those preventive measures, and the last most important step is 'make a plan'."
	Relatable to patient experiences	"I loved the individual stories and experiences. People like to relate to other people more than just reading facts."
		"[The patient experience where] he mentioned a family history of cancer three times and his brother finally got his colonoscopy, it sounds exactly like my experiences."
