# Supplementary material for: The Development and Evaluation of Novel Patient Educational Material for a Variant of Uncertain Significance (VUS) Result in Hereditary Cancer Genes
Source: Curr Oncol. 2024 Jun 16;31(6):3361–78. doi: 10.3390/curroncol31060256 (PMC11202617; doi:10.3390/curroncol31060256)
Supplement: Supplementary file 1 [file curroncol-31-00256-s001.zip › Supplemental Table S5.pdf]

Table S5. Constructive feedback organized by category and subcategory with supportive illustrative quotes and description of changes made.

Category	Subcategory	Illustrative Quotes	Changes Made
**Content**	More encouragement to share a VUS result	"I don't love the 'decided not to share' [section of the patient experiences] because I guess I don't have the same viewpoint that these people do and I recognize that it's an independent decision, it's personal."	Did not make changes, as materials are intentionally neutral because a VUS is not medically actionable.
**Visuals/ Audio**	Icon selection	"The clip art of the justice scales, that's really deterring because there's nothing fair or just about having to have this conversation."	Changed an icon from justice scales to a person shrugging.
**Usefulness**	Empower participants more	"Use more inclusive language of 'patients have indicated that this was a decision they had to wrestle with' or that they really thought about and found that these are some of the reasons why they might share [on the checklist of reasons to share the family history of cancer]."	Reformatted the checklist of reasons to share a family history to be in the 1st person.
		"Under 'Make a Plan' [on the cancer risks handout], maybe there could be a bullet point that is like… join a support group or figure out ways you can help support [someone with cancer], so again there is an action take away here."	Added action items/next steps to VUS Handout, planning guide for sharing with family, handouts on cancer risks.
	More guidance on who to share information with	"I saw something else earlier that listed how far [out in the family tree to share]… so maybe something [in the planning guide for sharing with family] would help tell you who you should be reaching out to. Do you want to go down to your second cousins, third cousins… so that might be helpful in a parenthesis or something to tell you how far to go."	Noted it is most important to share with your first-degree relatives like mother, father, sisters, brothers, and children.
