# Supplementary figures and images for: The Development and Evaluation of Novel Patient Educational Material for a Variant of Uncertain Significance (VUS) Result in Hereditary Cancer Genes
Source: Curr Oncol. 2024 Jun 16;31(6):3361–78. doi: 10.3390/curroncol31060256 (PMC11202617; doi:10.3390/curroncol31060256)

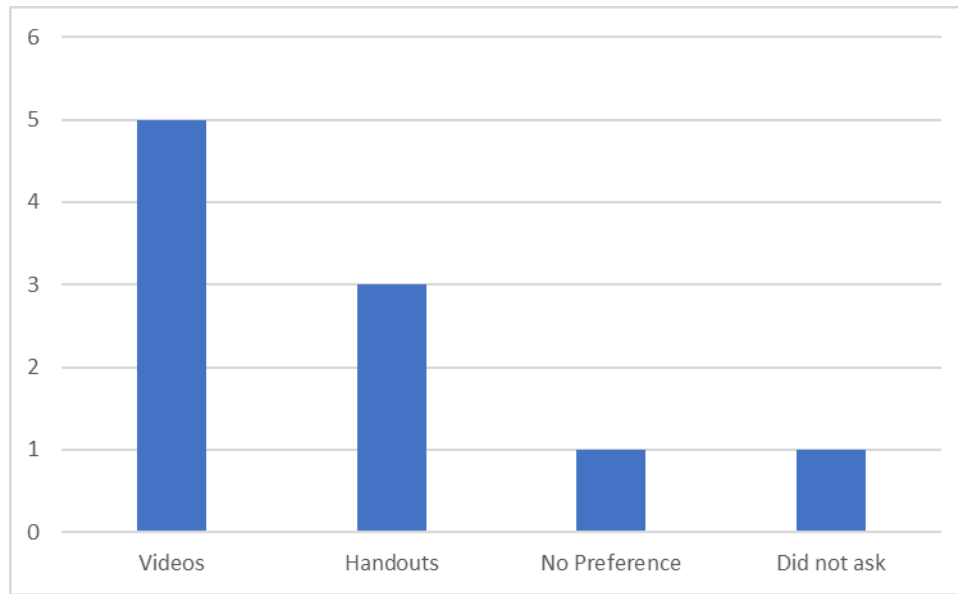

Supplemental Figure S1. Material format preference from second round of interviews.

Supplement: Supplementary file 1 [file curroncol-31-00256-s001.zip › Supplemental Figure S1.pdf]
